# Supplementary material for: Causal Relationship between Aging and Anorexia Nervosa: A White-Matter-Microstructure-Mediated Mendelian Randomization Analysis
Source: Biomedicines. 2024 Aug 16;12(8):1874. doi: 10.3390/biomedicines12081874 (PMC11351342; doi:10.3390/biomedicines12081874)
Supplement: Supplementary file 1 [file biomedicines-12-01874-s001.zip › Supplementary Figure/Figure S4/Singe SNP plot of MR analysis for TL to ubm-b-2049.pdf]

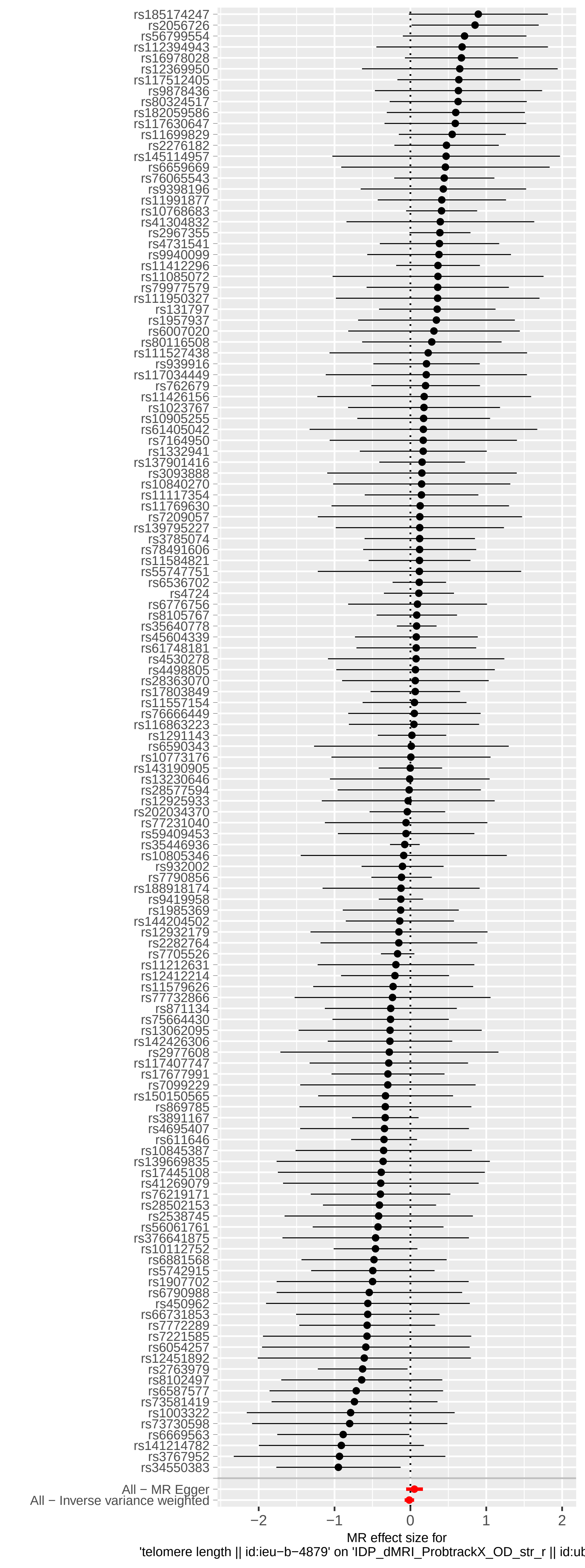
rs185174247
rs2056726
rs56799554
rs112394943
rs16978028
rs12369950
rs117512405
rs9878436
rs80324517
rs182059586
rs117630647
rs11699829
rs2276182
rs145114957
rs6659669
rs76065543
rs9398196
rs11991877
rs10768683
rs41304832
rs2967355
rs4731541
rs9940099
rs11412296
rs11085072
rs79977579
rs111950327
rs131797
rs1957937
rs6007020
rs80116508
rs111527438
rs939916
rs117034449
rs762679
rs11426156
rs1023767
rs10905255
rs61405042
rs7164950
rs1332941
rs137901416
rs3093888
rs10840270
rs11117354
rs11769630
rs7209057
rs139795227
rs3785074
rs78491606
rs11584821
rs55747751
rs6536702
rs4724
rs6776756
rs8105767
rs35640778
rs45604339
rs61748181
rs4530278
rs4498805
rs28363070
rs17803849
rs11557154
rs76666449
rs116863223
rs1291143
rs6590343
rs10773176
rs143190905
rs13230646
rs28577594
rs12925933
rs202034370
rs77231040
rs59409453
rs35446936
rs10805346
rs932002
rs7790856
rs188918174
rs9419958
rs1985369
rs144204502
rs12932179
rs2282764
rs7705526
rs11212631
rs12412214
rs11579626
rs77732866
rs871134
rs75664430
rs13062095
rs142426306
rs2977608
rs117407747
rs17677991
rs7099229
rs150150565
rs869785
rs3891167
rs4695407
rs611646
rs10845387
rs139669835
rs17445108
rs41269079
rs76219171
rs28502153
rs2538745
rs56061761
rs376641875
rs10112752
rs6881568
rs5742915
rs1907702
rs6790988
rs450962
rs66731853
rs7772289
rs7221585
rs6054257
rs12451892
rs2763979
rs8102497
rs6587577
rs73581419
rs1003322
rs73730598
rs6669563
rs141214782
rs3767952
rs34550383
All − MR Egger
All − Inverse variance weighted
−2
−1
0
1
2
MR effect size for
'telomere length || id:ieu−b−4879' on 'IDP_dMRI_ProbtrackX_OD_str_r || id:ub
